# Metabolomic insights into ultrasound-assisted fermentation of grape juice

**DOI:** 10.1016/j.ultsonch.2025.107537

**Published:** 2025-08-30

**Authors:** Liyue Fei, Dongsheng Zhang, Yu Li, Johane Johari Mkunga, Zinan Zhang, Chenglong He, Chunhui Shan, Muhammad Iqbal Choudhary, Xinquan Yang, Wenchao Cai

**Affiliations:** a Engineering Research Center of Storage and Processing of Xinjiang Characteristic Fruits and Vegetables, Ministry of Education, School of Food Science, Shihezi University, Shihezi, Xinjiang, China; b Key Laboratory of Processing and Quality and Safety Control of Specialty Agricultural Products (Co-construction by Ministry and Province), Ministry of Agriculture and Rural Affairs, School of Food Science, Shihezi University, Shihezi, Xinjiang, China; c School of Agriculture, Shihezi University, Shihezi, Xinjiang, China; d Office of the Party Committee of Xinjiang Production and Construction Corps, Urumqi, Xinjiang, China; e International Center for Chemical and Biological Sciences, University of Karachi, Karachi, Sandh, Pakistan; f Dar-es-salaam Institute of Technology, Dar-es-salaam, Tanzania

**Keywords:** Grape, Latic aicd bacteria, Fermentation, Ultrasound, Antioxidant activity, Metabolomics

## Abstract

Numerous studies have demonstrated that both lactic acid bacteria (LAB) fermentation and ultrasound-assisted fermentation can enhance the antioxidant activity of fruit juices; however, the effects of these two treatments on metabolites and antioxidant activity in grape juice (GJ) have yet to be investigated. Therefore, this study aimed to analyze the specific effects of LAB fermented grape juice (FGJ) and ultrasound-assisted fermented grape juice (UFGJ) on the antioxidant activity and metabolite production, while also conducting a preliminary investigation into the potential mechanisms underlying the antioxidant action of UFGJ using network pharmacology and molecular docking. The results indicated that UFGJ significantly enhanced the total phenolic content, total flavonoid content, and antioxidant activity of both FGJ and GJ (P < 0.001). Metabolomics analysis revealed highly significant differences in metabolite composition among the three groups (P < 0.001). The major differential metabolite classes and metabolic pathways of the FGJ versus GJ and UFGJ versus GJ groups were the same, with the latter group showing higher levels of shared metabolites. Thus, UFGJ does not alter the metabolic pathway of FGJ, but instead enhances its metabolic efficiency and increases metabolite enrichment. Correlation and network pharmacology analyses revealed that key antioxidant metabolites such as 2-hydroxycinnamic acid, gallic acid, and myricetin 3-rhamnoside primarily target core genes such as PPARG, EGFR, PTGS2, and PPARA, further regulating signaling pathways associated with chemical carcinogenesis-receptor activation, adherens junctions, and PPAR signaling pathway to exert antioxidant effects. Furthermore, molecular docking assays demonstrated that ursolic acid displaying the most stable binding to EGFR.

## 1 Introduction

Xinjiang is a traditionally advantageous grape production area in China, leading the country in both grape planting area and production. Among its regions, Turpan is the primary grape-producing area, boasting an extensive grape cultivation area of approximately 36,000 ha, earning it the title of the “World Grape Botanical Garden” both domestically and internationally. The ‘*Thompson Seedless*’ grape variety dominates the Turpan region, representing over 90 % of the total cultivated area. This variety is characterized by its thin skin, crisp flesh, juiciness, and sweetness, and possesses antioxidant properties that contribute to delaying aging and preventing cardiovascular diseases [63]. Consequently, it is highly favored by consumers [39]. However, challenges, including severe insect infestations in dried fruits, substantial berry shedding in fresh grapes, and poor transportation tolerance, have resulted in an oversupply of the ‘*Thompson Seedless*’ grape market, leading to significant wastage and hindering industrial development.

In recent years, the application of Latic aicd bacteria (LAB) in the production and processing of plant-based foods, particularly beverages, has emerged as a significant trend [14]. LAB fermentation not only imparts new flavors to plant-based beverages but also improves undesirable qualities of raw materials and enhances consumer acceptance [5,6,8,15]. Furthermore, LAB fermentation effectively enhances the functional properties of plant-based beverages, contributing to their antioxidant activity, anti-aging benefits, and mitigation of diabetes-related effects [64,78]. Although ultrasound is typically utilized for extracting functional ingredients and inactivating microbial cells, its application in fermentation can promote chemical and physical modifications of substrates, significantly altering the flavor profile of fermented plant-based beverages and improving their antioxidant activity and nutritional properties [2]. Therefore, ultrasound-assisted fermentation, characterized as an economical, environmentally friendly, efficient, safe, and non-toxic technology, is anticipated to enhance the metabolic efficiency of fermented plant-based beverages, thereby improving the overall fermentation efficiency of fruit juices. This advancement is significant for optimizing production space utilization and reducing energy consumption during production. Liu et al. [50] investigated the fermentation characteristics of hawthorn pulp fermented by LAB under five different ultrasonic power settings and found that low-power ultrasonication enhanced fermentation efficiency and quality, with 5 min of treatment at 360 W yielding the highest sugar, acid, and organoleptic quality indices. Hashemi et al. [34] demonstrated that ultrasound-assisted fermentation of Bakraei juice with LAB resulted in increased antioxidant activity correlated with the duration of ultrasound treatment. Sharma et al. [69] reported that LAB fermentation elevated the antioxidant activity of turmeric pulp from 67.49 % to 79.00 %, with further enhancement seen through ultrasound-assisted fermentation. Most existing literature on ultrasound-assisted fermentation of fruit and vegetable juices primarily explores fermentation performance and the antioxidant activity of specific functional substances, revealing a gap in research concerning the comprehensive changes in overall metabolites during ultrasound-assisted fermentation of these juices.

Metabolomics offers valuable insights into biological mechanisms and metabolic pathways by qualitatively and quantitatively analyzing small molecular metabolites across various samples and correlating differentially expressed metabolites with phenotypic changes [7]. This approach allows for the identification of key regulatory metabolites [36]. For example, Sun et al. [74] demonstrated that ultrasonic treatment increased triterpenoid levels in fermented Ganoderma lucidum juice by 34.96 %, highlighting its potential to enhance the metabolite profile of this juice. Chen et al. [17] found similar benefits from ultrasound in fermenting ginkgo almond juice, promoting the metabolism of phenolics, amino acids, and organic acids, ultimately enhancing its in vitro antioxidant activity. Grapes themselves are known for their potent antioxidant properties [29], which are further enhanced through LAB fermentation [19]. However, the majority of current studies on the antioxidant properties of LAB fermented grape juice (FGJ) adhere to the traditional “single component-single target” model, which inadequately represents the human body’s antioxidant mechanisms [35]. Therefore, this study aims to explore a multi-component and multi-target interaction model to elucidate the potential antioxidant mechanisms of ultrasound-assisted fermented grape juice (UFGJ). Network pharmacology facilitates a systematic analysis of multi-target synergistic effects by constructing a biological network connecting “drug-target-disease,” offering a comprehensive perspective on target components and assisting in clarifying the molecular mechanisms underlying the antioxidant actions of metabolite molecules [37]. For instance, Lazzara et al. [43] enabled rapid prediction of potential mechanisms of action via drug-target-disease network mappings. Molecular docking optimizes the three-dimensional structures of proteins and small molecule drugs, ultimately determining binding affinities through binding energy assessments [37]. Wang et al. [84] utilized untargeted metabolomics to explore metabolic variations in different Gastrodia elata Blume samples, identifying seven core antioxidant metabolites, including isoreserpin and phytosphingosine, and their potential targets such as GAPDH, EGFR, and SRC, which may exert antioxidant effects through pathways like PI3K-Akt and IL-17. These studies deepen the understanding of phytochemistry and highlight the therapeutic potential of natural antioxidants in fruits and vegetables against oxidative stress-related diseases, while providing new insights into the molecular mechanisms of key antioxidant compounds within the context of fruit and vegetable fermentation.

This research investigates the effects of ultrasound technology on LAB fermentation of ‘*Thompson Seedless*’ GJ by examining the total phenolic content (TPC), total flavonoid content (TFC), and in vitro antioxidant capacity of samples from grape juice (GJ), FGJ, and UFGJ. Initially, the impact of ultrasound-assisted fermentation technology on the functional components of the FGJ was assessed. Subsequently, a non-targeted metabolomics approach was employed to evaluate the metabolites across the three groups, analyzing the compounds responsible for quality differences among the juices. Finally, through network pharmacology and molecular docking, we predicted target genes and pathways associated with key differential metabolites related to antioxidant activity, simulating and verifying the molecular interactions between core metabolites and target proteins, thereby elucidating the potential antioxidant mechanisms of UFGJ metabolites. The findings contribute to our understanding of the material basis for differences between FGJ and UFGJ, facilitating further exploration of the impact of ultrasound-assisted fermentation at the metabolite level in ‘*Thompson Seedless*’ grapes. Additionally, this research presents innovative approaches for GJ fermentation processes, providing a theoretical foundation for UFGJ.

## 2 Materials and methods

### 2.1 Sample Collection

In the summer of 2024, we purchased ‘*Thompson Seedless*’ grapes from the farmers’ market in Shihezi, Xinjiang, China, and transported them back to the laboratory in a temperature-controlled sampling box equipped with ice packs. The selected grapes were full, displaying no signs of mold or disease. After washing with deionized water, the grapes were drained, pulped using a pulper, divided into sterile bottles, and stored in a refrigerator at −20℃ for subsequent experimental analyses.

### 2.2 Preparation of ultrasound-assisted fermented grape juice

An appropriate amount of previously preserved GJ was thawed. After complete thawing, the juice was sterilized by heating, allowed to cool to room temperature, and a portion was saved to form GJ group. Then, 50 µL of the conserved bacterial solution of LP-CWC-Zao was transferred into a test tube containing 5 mL of MRS liquid medium for activation, followed by a 24-hour incubation. Subsequently, 500 µL of the activated bacterial solution was transferred into a conical flask containing 50 mL of MRS liquid medium and incubated for an additional 24 h to obtain the fermentation broth [4]. LP-CWC-Zao is a strain of lactic acid bacteria (LAB) suitable for fruit and vegetable fermentation, previously selected by our research team [9]. The fermentation broth was then inoculated into the sterilized GJ at a 2 % (V/V) inoculum under aseptic conditions, mixed thoroughly, and placed in a sterile biochemical incubator for anaerobic fermentation at a constant temperature of 37℃ for 36 h to obtain the FGJ group. After this, the UFGJ group was created using a CNC ultrasonic cleaner KQ-800DE (Kunshan Ultrasonic Instrument Co., Ltd., Jiangsu, China) operated at an ultrasonic frequency of 40 kHz and a power of 320 W for 10 min at the 12th, 24th, and 36th hours of fermentation. Five samples of grapes were taken from each group, totaling 15 samples, which were subsequently stored in a −20℃ refrigerator for subsequent experimental analysis.

Pawar and Rathod [61] concluded that low-frequency ultrasound, specifically below 100 kHz, is beneficial for microbial cells, and that low-frequency, low-energy ultrasound is more effective for enhancing the quality of fermented products. Liu et al. [49] employed various ultrasound powers at 40 kHz (0 W, 300 W, 360 W, 420 W, and 540 W) to investigate the impact of ultrasound treatment on the fermentation performance and quality of lactic acid bacteria fermented hawthorn pulp. They discovered that an ultrasound power of 360 W maximized both the fermentation performance and quality of the fermented juice, whereas high-power ultrasound (540 W) diminished both the fermentation performance and quality. Therefore, we selected a frequency of 40 kHz and a power level of 320 W for processing the FGJ.

### 2.3 Bioactive compounds and antioxidant activity analysis

TPC and TFC were determined using the methods of Cai et al. [12] with slight modifications. The methods described by Cai et al. [9] were similarly adapted for assessing the ABTS^+^ and DPPH radical scavenging rates of the samples. Five biological replicates and three technical replicates were used for the determination of bioactive compounds and antioxidant activity.

### 2.4 UHPLC-Q-TOF-MS analysis

Metabolomics determination was conducted using a UHPLC-Q Exactive HF-X system (ThermoFisher Scientific, USA). Chromatographic separation was performed on an ACQUITY UPLC HSS T3 column (100 mm × 2.1 mm i.d., 1.8 µm; Waters, Milford, USA). The samples were first separated by liquid chromatography to achieve component separation, after which mass spectra were collected based on mass-to-charge ratio. Qualitative and quantitative results were derived by analyzing the mass spectral data of the samples. Sample pretreatment, instrument configuration, and data processing were conducted in accordance with our previous studies [10,37]. To ensure the reliability of the metabolomics data, each sample group included five biological replicates, resulting in a total of 15 metabolomics samples.

### 2.5 Network pharmacology analysis

The SMILES formulas of differential metabolites were retrieved from the PubChem database and submitted to the Swiss Target Prediction database, screening for valid targets with “Possibility > 0.2”. The term “Resistant to Oxidation” was used as a search criterion in the GeneCards database to identify targets with a score ≧ 5 and to eliminate duplicates. A Venn diagram was generated using the Venny 2.1 tool to establish an intersection target library. Cytoscape 3.9.1 software was employed to create both differential metabolite-metabolite-target networks and protein–protein interaction (PPI) network maps. Gene Ontology (GO) functional enrichment analysis and Kyoto Encyclopedia of Genes and Genomes (KEGG) pathway enrichment analysis were performed on the core targets using R software.

### 2.6 Molecular docking

The 2D structures of the differential metabolites were downloaded from the PubChem database. Molecular docking and binding energy calculations were performed using AutoDockTools-1.5.7 software, with visualization of promising binding results conducted via PyMOL-2.0 software, focusing on the lowest binding energy that included hydrogen bonds as the docking result [35,37].

### 2.7 Statistical analysis

All determinations were performed in triplicate, with results expressed as mean ± standard deviation. Statistical differences were analyzed using one-way ANOVA. R software (version 4.1.1, https://www.r-project.org/) was utilized for Permutational Multivariate Analysis of Variance (PERMANOVA), Principal Component Analysis (PCA), Partial Least Squares Discriminant Analysis (PLS-DA), and correlation analysis. All graphs were produced using R software and Origin software.

## 3 Results and Discussion

### 3.1 Effect of UFGJ on the antioxidant activity

**Fig. 1:**
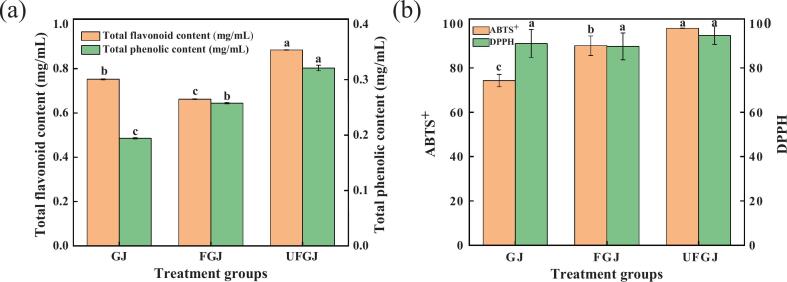
(a) Total phenolic content and total flavonoid content; (b) ABTS^+^, DPPH free radical scavenging capacity.

Polyphenols are among the most significant secondary metabolites in grapes, contributing significantly to their antioxidant activity [39]. Therefore, in this study, we first measured the TPC of GJ samples from different treatment groups and found a highly significant difference among the three groups (P < 0.001). As illustrated in Fig. 1a, UFGJ exhibited the highest TPC (0.321 ± 0.005 mg/mL), followed by FGJ at 0.258 ± 0.001 mg/mL, and GJ at 0.194 ± 0.001 mg/mL. This indicates that LAB fermentation promotes the solubilization of polyphenols from GJ, with ultrasound treatment enhancing this process. Kuerban et al. [42] utilized four species of LAB to ferment GJ and noted a significant increase in TPC during fermentation, which aligns with our findings. Sheng et al. [70] suggested that the increase in TPC due to LAB fermentation may be related to changes in the acidic environment caused by LAB growth and microbial hydrolysis. Specifically, the pH changes during fermentation can affect the structure of phenolic compounds, allowing for greater detection of these compounds [28]. Moreover, the production of glycosidases or esterases by LAB may lead to the cleavage and acidification of glycosides and esters bound to phenolic acids, converting bound phenols to free phenols in GJ, ultimately increasing the TPC [42]. Furthermore, Blaszak et al. [3] found that ultrasound treatment at a frequency of 40 kHz significantly increased TPC in GJ, which is consistent with our study’s results. This enhancement may be attributed to ultrasound promoting lactic acid biosynthesis and free amino acid catabolism by LAB, modulating phenolic derivatives in GJ [80].

Flavonoids are the most abundant class of secondary metabolites in grapes, protecting them from ultraviolet radiation, pests, and diseases while playing a crucial role in their color, flavor, and nutritional value [32]. In this study, the TFC of GJ samples from different treatment groups was further assessed, revealing highly significant differences among the three groups (P < 0.001). As shown in Fig. 1a, UFGJ had the highest TFC (0.884 ± 0.001 mg/mL), followed by GJ (0.752 ± 0.002 mg/mL), and FGJ (0.662 ± 0.002 mg/mL). This suggests that fermentation promotes the consumption of flavonoids in GJ while ultrasound significantly increases TFC. Wu et al. [85] observed a gradual decrease in TFC during LAB fermentation, which is consistent with our findings. This phenomenon may result from the depolymerization of high molecular weight phenolic compounds by (poly)phenol oxidase in LAB strains, thereby reducing TFC in FGJ [20]. Additionally, since TFC in UFGJ was higher than that in GJ and FGJ, we can hypothesize that the efficiency of ultrasound treatment in synthesizing TFC surpassed the efficiency of fermentation in depleting TFC in GJ. Hence, ultrasound treatment can accelerate TFC synthesis in GJ and assist LAB in fermenting ‘*Thompson Seedless*’ GJ.

Polyphenols and flavonoids in grapes have important physiological and health benefits, with numerous studies indicating their diverse biological activities, including anti-free radical damage, anti-inflammatory, antioxidant, anti-radiation, and cardiovascular protective effects [41,60]. Therefore, we conducted ABTS^+^ and DPPH antioxidant assays to evaluate the antioxidant activity of GJ from different treatment groups. Notably, while the DPPH free radical scavenging capacity did not show significant differences among the treatment groups (P > 0.05), the ABTS^+^ free radical scavenging capacity exhibited highly significant differences among the three groups (P < 0.001). As shown in Fig. 1b, the highest ABTS^+^ activity was observed in UFGJ (97.935 ± 0.001 %), followed by FGJ (90.068 ± 0.044 %) and GJ (74.323 ± 0.028 %). This indicates that LAB fermentation and ultrasound-assisted fermentation significantly enhance the ABTS^+^ activity of GJ, with ultrasound treatment providing a more substantial increase in antioxidant activity compared to fermentation alone. Hashemi et al. [34] also reported that ultrasound-assisted fermentation increased the antioxidant activity of fruit juices. Sheng et al. [70] hypothesized that the increased ABTS^+^ activity could depend on structural changes in phenolic hydroxyl groups on the benzene ring; thus, the ABTS^+^ trend may correlate with TPC. The results of this study confirm their hypothesis, with a similar trend observed in TPC and ABTS^+^ values. Although DPPH did not show significant differences among the groups, it remained highest in the UFGJ. Hence, ultrasound-assisted fermentation notably enhances the antioxidant activity of GJ more effectively than fermentation alone.

In summary, the content of functional substances and antioxidant activity (Fig. 1) across all treatment groups demonstrated that the three different treatments significantly impacted TPC, TFC, and ABTS^+^ (P < 0.001), with UFGJ exhibiting the highest TPC, TFC, and ABTS^+^, followed by FGJ and GJ. This study utilized short-term intermittent ultrasound treatment, which can form temporary and reversible pores in microbial cell membranes, improving cell membrane permeability, accelerating intracellular and extracellular mass transfer, and promoting the metabolism of intracellular fermentation-related substances, thereby enhancing the fermentation efficiency of LAB [34]. Consequently, it was concluded that ultrasound-assisted fermentation treatments can provide greater benefits in terms of functional substance content and antioxidant activity compared to standard fermentation treatments.

### 3.2 Effect of UFGJ metabolites

**Fig. 2:**
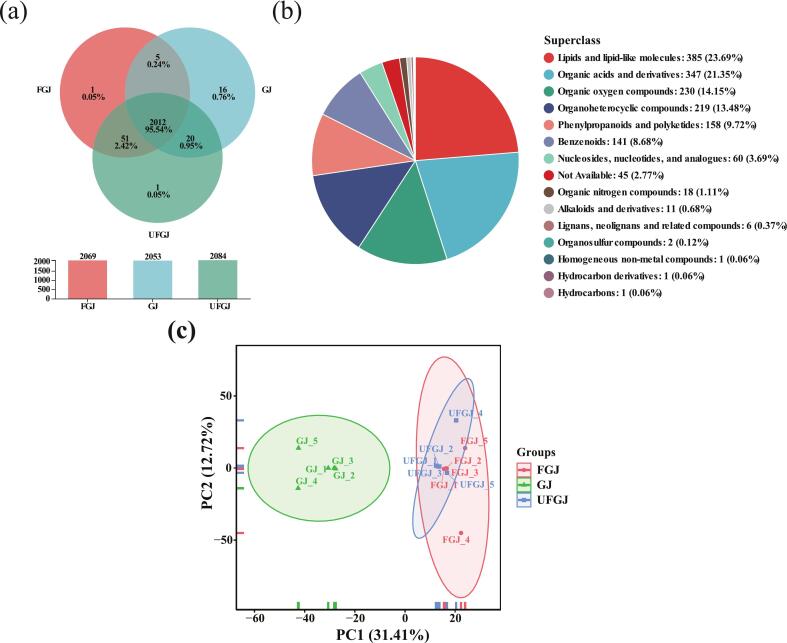
(a) Veen diagram; (b) HMDB classification of total metabolites and (c) PCA score plots of metabolites from three treatment groups of grape samples.

Given the significant differences in both the content of functional components and antioxidant properties among GJ samples from various treatments, we aimed to further investigate the impact of ultrasound-assisted fermentation on the metabolite composition of ‘*Thompson Seedless*’ GJ and its patterns of change. The number of shared and unique metabolites across different groups was analyzed using a Venn diagram (Fig. 2a), revealing a total of 2,106 detected metabolites. Among these, GJ contained 2,053 metabolites, FGJ had 2,069 metabolites, and UFGJ contained 2,084 metabolites. The number of metabolites shared among the three treatment groups was 2,012, and those shared between GJ and FGJ, FGJ and UFGJ, and GJ and UFGJ were 2,017, 2,063, and 2,032, respectively. Each treatment group also had specific metabolites; GJ had 16 specific metabolites, primarily consisting of amino acids and their derivatives, such as Leucyl-Leucine and Leucyl-Aspartate. Thus, we hypothesize that the amino acid metabolic pathway may play a critical role during GJ fermentation. Amino acids serve as important precursors for flavor compounds such as higher alcohols in FGJ and UFGJ, thereby contributing to the flavor profile in FGJ. Zhao et al. [96] discovered that L-phenylalanine is converted to phenylethanol mainly through the Ehrlich pathway, facilitated by transaminase, decarboxylase, and dehydrogenase, which gives a rosy aroma to Junjube Fruit Wine. FGJ and UFGJ identified only one specific metabolite each: N-Octanoylsphingosine and 1,1-Diphenyl-2-Picrylhydrazine, respectively.

We detected and annotated a total of 1,625 metabolites from the HMDB public database for all treatment groups (Fig. 2b). These metabolites can be categorized into ten groups: 385 lipids and lipid-like molecules (23.69 %), 347 organic acids and derivatives (21.35 %), 230 organic oxygen compounds (14.15 %), 219 organoheterocyclic compounds (13.48 %), 158 phenylpropanoids and polyketides (9.72 %), 141 benzenoids (8.68 %), 60 nucleosides, nucleotides, and analogues (3.69 %), 18 organic nitrogen compounds (1.11 %), 11 alkaloids and derivatives (0.68 %), and 56 other organic compounds (3.45 %).

### 3.3 PCA and PLS-DA analysis

**Fig. 3:**
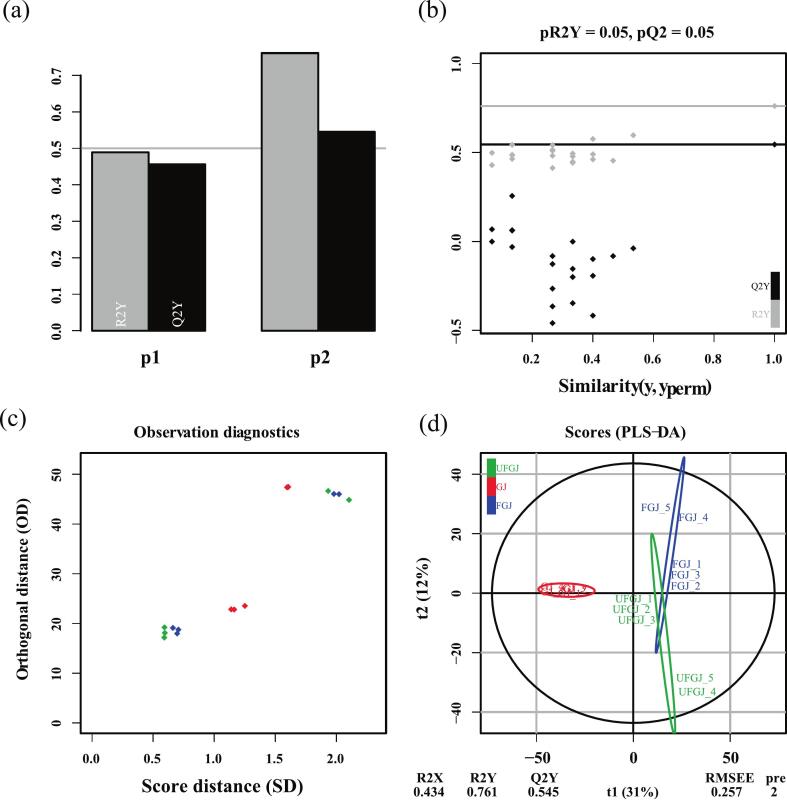
(a) OPLS-DA model overview, (b) significance diagnostics, (c) observation diagnostics, and (d) score plots for grapes of three treatment groupst.

To investigate the variability of GJ metabolites among the treatment groups, we analyzed the metabolites from the three groups using principal component analysis (PCA). As shown in (Fig. 2c), GJ was clearly differentiated from FGJ and UFGJ, whereas FGJ and UFGJ showed a significant overlap, thus the metabolite composition of the two groups was considered to be more similar. However, the antioxidant results showed that UFGJ was significantly higher than FGJ and GJ, so in order to clarify the metabolites that caused the differences between the three treatment groups, PLS-DA was conducted. Fig. 3 illustrates that both models contained sufficient orthogonal components, passed substitution tests, and were stable, reliable, and free from overfitting. PLS-DA again demonstrated the similarity in the metabolite composition of FGJ and UFGJ, and it is hypothesized that the difference in antioxidant results may stem more from the difference in metabolite content between the two groups than from the difference in type, and thus further analysis in terms of specific content of metabolites is needed.

### 3.4 Analysis of differential metabolites in UFGJ

To identify specific differential metabolites that can effectively represent and distinguish between the various treatment groups, metabolite analysis was conducted with VIP > 1.00 and P < 0.05 as screening criteria (Tables S1–S3). A total of 559 differential metabolites were identified among the three treatment groups, with the counts of differential metabolites being 468 for FGJ vs. GJ, 166 for UFGJ vs. FGJ, and 463 for UFGJ vs. GJ.

In the FGJ vs. GJ comparison (Table S1), 227 compounds were up-regulated while 241 were down-regulated. The differential metabolites were primarily organic acids and derivatives, lipids and lipid-like molecules, and organoheterocyclic compounds. Notable metabolites included ketoleucine, proline betaine, 1-aminocyclopropanecarboxylic acid, *sn*-*glycero*-3-phosphocholine, azucubin, mevalonolactone, indole-3-acetaldehyde, isoeriocitrin, glucuronolactone, and inulobiose all of which exhibited high VIP values. Ketoleucine, found in small amounts in bananas and asparagus, contributes fruity, sweet flavors. Proline betaine, an alkaloid isolated from motherwort and other Chinese herbs, has been well-documented for its benefits in preventing and treating cardiovascular and central nervous system diseases by modulating various signaling pathways and molecular targets [46]. 1-Aminocyclopropanecarboxylic acid is a cyclic amino acid commonly found in pears and apples; it is synthesized from methionine, eventually leading to ethylene production. Sn-*glycero*-3-phosphocholine (GPC) serves as a biosynthetic precursor of acetylcholine, a key neurotransmitter, and can cross the blood–brain barrier [45]. GPC not only helps prevent cognitive decline due to aging and enhances memory but also stimulates central functions by promoting growth hormone secretion, which may mitigate Alzheimer’s disease [59].

Aucubin is a cyclic enol ether terpene known for its anti-inflammatory, antioxidant, and neuroprotective effects [40]. Gao et al. [31] discovered that aucubin can enhance the anti-tumor activity of cisplatin by inhibiting PD-L1 expression in hepatocellular carcinoma. Mevalonolactone is a crucial intermediate in cellular biosynthesis, serving as a precursor to many vital biologically active aliphatic compounds, including cholesterol, steroids, and bile acids. Chen et al. [16] found that mevalonolactone could improve stratum corneum barrier function by elevating PPARβ/δ levels and facilitating fatty acid transport to lamellar granules, ultimately increasing intercellular lipid levels. Indole-3-acetaldehyde, a product of tryptophan metabolism, has demonstrated anti-tumor effects through direct action on tumor cells and immunomodulation [23]. Isoeriocitrin, the primary flavonoid in lemons, exhibits strong antioxidant, anti-tumor, and anti-allergic properties, particularly in lowering blood lipids, providing antidiabetic effects, and protecting cells from specific metabolic-induced damage by activating the Nrf2/HO-1 antioxidant enzyme system and inhibiting inflammatory factors like NF-κB, TNF-α, IL-1β, and IL-6 [90].

In the UFGJ vs. GJ comparison (Table S2), 231 compounds were up-regulated and 232 compounds down-regulated, with differential metabolites primarily being organic acids and derivatives, lipids and lipid-like molecules, organoheterocyclic compounds, and organic oxygen compounds, consistent with the FGJ vs. GJ analysis. Among them were L-phenylalanine, L-glutamine, cyasterone, cyclic adp-ribose, myricetin, proline betaine, ketoleucine, aucubin, glucuronolactone, mevalonolactone, indole-3-acetaldehyde, inulobiose, *sn*-glycero-3-phosphocholine, and other differential metabolites exhibiting high VIP values. Notably, many of these metabolites were also present in the FGJ vs. GJ group, suggesting that ultrasound treatment does not substantially alter the metabolite composition of FGJ.

L-phenylalanine is an aromatic amino acid widely utilized across food, medicinal, and chemical industries due to its significant biological functions. Cyasterone, extracted from hyssop root, has shown positive effects on treating osteoporosis and osteoporotic fractures and acts as a natural inhibitor of the epidermal growth factor receptor, marking it as a promising anticancer drug [75]. Cyclic ADP-ribose is an intracellular calcium mobilizer that plays a key role in energy storage and homeostasis. Metabolomics analyses suggest its involvement in regulating adipocyte differentiation in mice [76]. Myricetin, a flavonoid compound found in numerous plants, exhibits various biological activities, including anti-inflammatory, antibacterial, anti-tumor, and anti-obesity effects [73], and has recently demonstrated a crucial role in treating liver disease [18].

In the UFGJ vs. FGJ comparison (Table S3), 111 compounds were up-regulated and 55 compounds down-regulated, with differential metabolites primarily comprising lipids and lipid-like molecules, organic acids and derivatives, and phenylpropanoids and polyketides. Additionally, the FGJ vs. GJ and UFGJ vs. GJ groups shared a total of 435 differential metabolites, including kanokoside A, L-phenylalanine, L-glutamine, 3-methylcatechol, betonicine, 2-hydroxycinnamic acid, traumatic acid, cinnamic acid, glucuronolactone, and other compounds, with the total number of shared compounds in UFGJ exceeding that in FGJ. Therefore, ultrasound treatment does not disrupt or inhibit the metabolic properties of LAB in GJ; rather, it enhances metabolic efficiency and increases metabolite content, aligning with Hashemi et al. [34].

Furthermore, 29 metabolites unique to ultrasound-assisted fermentation were identified by comparing the differential metabolites of the UFGJ vs. GJ group with those of the FGJ vs. GJ group (Table S4). Noteworthy metabolites include araloside A, bergenin, ursolic acid, jangomolide, 3,3-dimethylglutaric acid, and fraxin. Araloside A, an oleanolic acid-type pentacyclic triterpenoid saponin, is a major active component of the wild medicinal and edible plant Aralia elata (Miq.) Seem [79], exhibiting antioxidant, pro-apoptotic, and anti-inflammatory effects with promising therapeutic potential for inflammatory conditions such as rheumatoid arthritis and ethanol-induced gastric ulcers [54]. Bergenin is a natural isocoumarin analog found in various plants, possessing pharmacological activities such as anti-inflammatory, antioxidant, anti-malarial, and immunomodulatory effects [88]. It has been shown to protect cardiomyocytes from apoptosis through the SIRT1 pathway [51]. Fraxin is a coumarin-like compound with anti-inflammatory, antimicrobial, and antioxidant properties [62], shown to inhibit neuroinflammation and oxidative stress via the PPAR-γ/NF-κB pathway, thereby alleviating cerebral ischemia–reperfusion injury [89]. Recent studies have indicated that fraxin suppresses apoptosis and inflammation by inhibiting OCT3-mediated activation of the FGF2/NF-κB pathway, thereby reducing inflammation in murine oral lichen planus tissues [62]. Hence, ultrasound-assisted fermentation is capable of producing new substances with antioxidant activity, increasing the nutritive and functional constituents of ‘*Thompson Seedless*’ GJ, which may explain why the antioxidant activity of the UFGJ group was higher than that of FGJ.

### 3.5 Analysis of KEGG enrichment pathways for differential metabolites in UFGJ

Fruit and vegetable juice fermentation is a complex biochemical process that involves numerous substances and reactions governed by multiple interacting regulatory mechanisms [11,13]. Comprehensively assessing this process requires more than just examining changes in the content of a single substance. Therefore, to investigate the effects of different treatments on the metabolites in ‘*Thompson Seedless*’ GJ, in-depth analyses of the metabolic pathways are necessary. Bioinformatics analysis of the previously screened differential metabolites in the KEGG database reveals the roles and interrelationships of these metabolites during different treatments and provides insight into the metabolic pathways involved, allowing for an analysis of how different treatments affect ‘*Thompson Seedless*’ GJ.

**Fig. 4:**
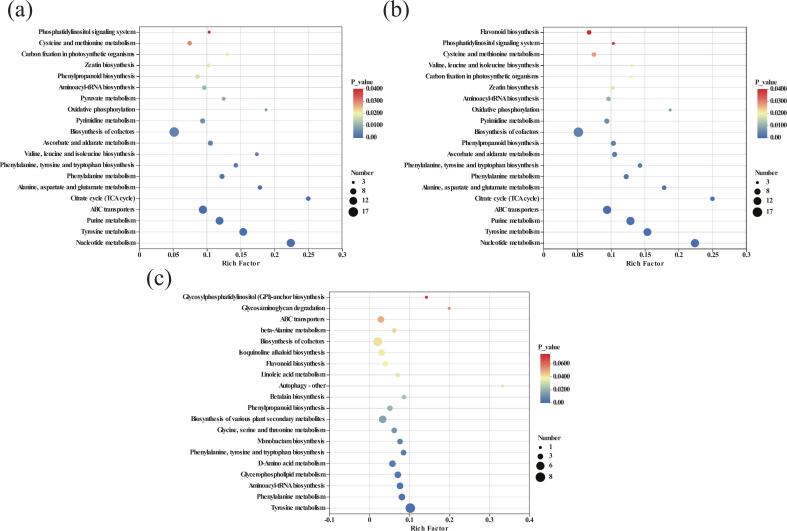
Plot of KEGG enrichment analysis of three groups of differential metabolites.

As shown in Table S5, the functional annotation of the FGJ vs. GJ group indicates that a total of 76 metabolic pathways were integrated, encompassing 125 metabolites. Among these, 38 metabolic pathways were down-regulated, including the citrate cycle (TCA cycle), ABC transporters, pyrimidine metabolism, and aminoacyl-tRNA biosynthesis, while 28 pathways were up-regulated, including alanine, aspartate, and glutamate metabolism, and phenylalanine, tyrosine, and tryptophan biosynthesis. The TCA cycle serves as the final metabolic pathway for the three major nutrients (sugars, lipids, and amino acids) and is a hub for the interconversion of various organic substances [27]. Therefore, the downregulation of the TCA cycle is significant, as it also affects the levels of citric acid and isocitric acid. Additionally, LAB convert L-malic acid to fumaric acid through enzyme-catalyzed reactions [52], which is consistent with the observed downregulation of L-malic acid and the up-regulation of fumaric acid. Fig. 4 demonstrates the 20 metabolic pathways that were highly enriched in the FGJ vs. GJ group, where the top five pathways with the highest degree of enrichment were biosynthesis of cofactors, ABC transporters, tyrosine metabolism, purine metabolism, and nucleotide metabolism.

The biosynthesis of cofactors involves compounds that bind to proteins, essential for their normal catalytic function, making their production important for metabolic reactions in the body [58]. Most ABC transporter proteins can bind ATP and release energy by hydrolyzing it, regulating the transmembrane transport of substances. Thus, ABC transporters play a vital role in the transport and metabolism of key compounds during juice fermentation [53], including L-aspartic acid, L-phenylalanine, and erythritol, etc. L-Phenylalanine serves as a crucial precursor for the synthesis of secondary metabolites such as coumarins, flavonoids, phenolics, and lignans [66]. Consequently, downregulation of L-phenylalanine alongside upregulation of quercetin and isoquercitrin can be observed.

In the tyrosine metabolism pathway, L-tyrosine and L-phenylalanine are catalyzed by aspartate aminotransferase to generate L-glutamate [67]). L-Tyrosine can be catalyzed by aromatic aldehyde synthase (AAS), resulting in the formation of 4-hydroxyphenylglyoxal (4-HPAA) through the deamidation of the amino group [22], and can also convert to L-DOPA via polyphenol oxidase catalysis. Purine metabolism is closely related to nucleotide metabolism; the synthesis of substances for nucleotide metabolism depends on the smooth progression of purine metabolism [53], meaning these two pathways share certain metabolites, such as deoxyuridine, L-glutamine, and uridine. LAB can prevent or treat hyperuricemia by degrading purines and significantly reducing uric acid levels in the body [81], which may also explain the downregulation of guanine and adenine in FGJ.

As shown in Table S6, the functional annotation of the UFGJ vs. GJ group indicated that a total of 74 metabolic pathways were integrated, covering 125 metabolites. Among these, 38 metabolic pathways were downregulated, including the TCA cycle, ABC transporters, pyrimidine metabolism, aminoacyl-tRNA biosynthesis, and phenylpropanoid biosynthesis. Conversely, 29 metabolic pathways were upregulated, including alanine, aspartate, and glutamate metabolism, phenylalanine, tyrosine, and tryptophan biosynthesis, and glutathione metabolism. Fig. 4 illustrates the 20 metabolic pathways highly enriched in the UFGJ vs. GJ group, where amino acid biosynthesis, secondary metabolite biosynthesis, and carbohydrate metabolic pathways play critical roles in the LAB fermentation quality of ‘*Thompson Seedless*’ GJ. Notably, all 74 metabolic pathways in the UFGJ vs. GJ group are included in the 76 pathways in the FGJ vs. GJ group, with the five most highly enriched pathways consistent in both groups, indicating that ultrasound-assisted fermentation does not alter the metabolic pathways of LAB in FGJ; rather, it increases the degree of metabolite enrichment. This finding aligns with the physicochemical properties and the differential metabolite results, reaffirming that ultrasound treatment does not disrupt or inhibit LAB’s metabolic properties in GJ, but rather accelerates metabolic efficiency and enhances metabolite content.

As shown in Table S7, the functional annotation of the UFGJ vs. FGJ group revealed a total of 52 integrated metabolic pathways encompassing 48 metabolites. Among these, eight metabolic pathways were downregulated, including flavonoid biosynthesis, anthocyanin biosynthesis, and flavone and flavonol biosynthesis, while 39 metabolic pathways were upregulated, including phenylalanine metabolism, phenylalanine, tyrosine, and tryptophan biosynthesis, and phenylpropanoid biosynthesis. Flavonoid biosynthesis, anthocyanin biosynthesis, and flavone and flavonol biosynthesis are pathways associated with flavonoid metabolism, involving metabolites such as cyanidin, pelargonidin, L-epicatechol, and kaempferol-3-O-glucoside. Studies have shown that these compounds exhibit anti-inflammatory, antioxidant, cardiovascular enhancement, immune-boosting, and aging-delaying functions [24]. Notably, although these metabolites were downregulated, the physicochemical properties indicate that the total flavonoid content was higher in UFGJ than in FGJ. This discrepancy could arise from the fact that not all detected flavonoids were annotated in KEGG pathways or that non-targeted metabolomics did not capture all flavonoids present in the samples. Fig. 4 highlights the 20 metabolic pathways highly enriched in the UFGJ vs. FGJ group, with tyrosine metabolism, phenylalanine metabolism, aminoacyl-tRNA biosynthesis, glycerophospholipid metabolism, and D-amino acid metabolism being the five pathways with the highest enrichment.

In the phenylalanine metabolic pathway, L-phenylalanine converts to L-tyrosine primarily through the action of phenylalanine hydroxylase [30]. Both L-phenylalanine and L-tyrosine are bitter amino acids, suggesting that this metabolic pathway contributes to flavor formation in FGJ. Furthermore, phenylalanine can be converted to cinnamic acid by phenylalanine aminotransferase, which subsequently generates o-hydroxycinnamic acid through the action of cinnamate-3-hydroxylase (C3H) [83], leading to increased levels of 2-hydroxycinnamic acid in the UFGJ group. Many aminoacyl-tRNA synthetases (aaRS) are involved in the biosynthesis of microbial secondary metabolites within the aminoacyl-tRNA biosynthesis pathway [57]. These aaRS are responsible for pairing the correct amino acid with its corresponding tRNA, thereby determining the genetic code [77]. However, Zhao et al. [92] found that LAB produces enzymes, such as glutaminase, that degrade proteins and biosynthesize amino acids, resulting in upregulation of all amino acids annotated in the aminoacyl-tRNA biosynthesis pathway. Glycerophospholipid metabolism promotes the digestion, absorption, and transport of lipids, constituting an essential aspect of lipid metabolism.

Consequently, the metabolic pathways in the FGJ vs. GJ group were primarily enriched in pathways related to amino acid metabolism, carbohydrate metabolism, and energy metabolism. The degradation of amino acids resulted in an increase in secondary metabolites such as flavonoids, phenolics, and organic acids in GJ, with upregulation observed in succinic acid, fumaric acid, and 4-hydroxyphenylacetaldehyde. The UFGJ vs. GJ group showed enrichment in secondary metabolite biosynthesis, amino acid metabolism, and lipid metabolism, leading to significant accumulation of small-molecule amino acids (e.g., L-tyrosine, L-phenylalanine) by enhancing LAB’s ability to degrade proteins, which further facilitated the synthesis of secondary metabolites, including 2-hydroxycinnamic acid, gallic acid, and p-coumaraldehyde, all of which were upregulated. Thus, LAB fermentation preferentially utilizes free amino acids in GJ to produce various secondary metabolites, while ultrasound-assisted LAB fermentation enhances the catabolism of large proteins in GJ, resulting in the production of smaller amino acids and secondary metabolites. Consequently, ultrasound-assisted fermentation generates a greater quantity of metabolites, aligning with findings from Sun et al. [74]. These findings may inform the design of microbial consortia for flavor modulation in future fermented functional beverages. Furthermore, the complex metabolic processes involved in ultrasound-assisted LAB fermentation of ‘*Thompson Seedless*’ GJ indicate that the quality of the fermented juice results from the co-regulation of various compounds, enzymes, and metabolic pathways. Thus, further exploration of the intrinsic mechanisms underlying the formation of fermented juice quality in ultrasound-assisted fermentation is warranted, ideally in combination with transcriptomics and proteomics.

### 3.6 Screening of key antioxidant metabolites of UFGJ

**Fig. 5:**
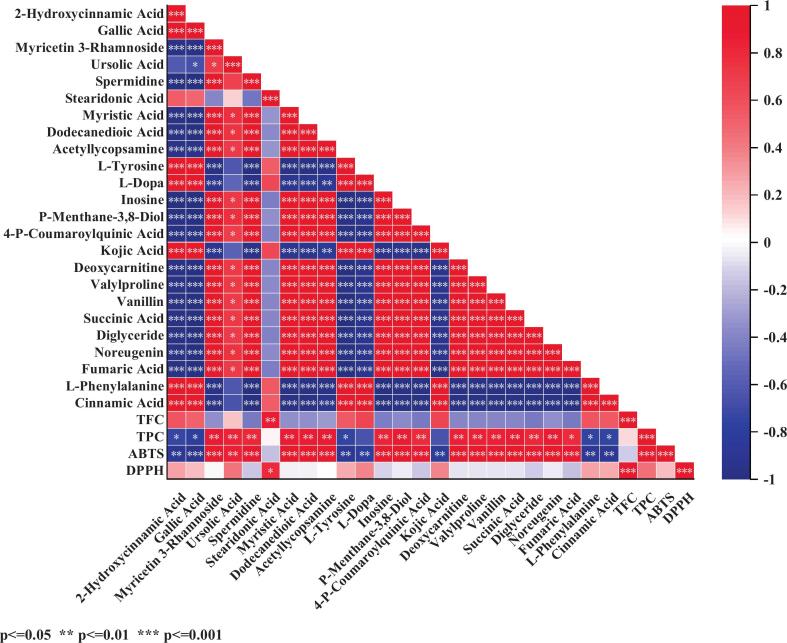
Correlation analysis of antioxidant indexes of some differential metabolite.

Based on the conclusions regarding the physicochemical properties, it was determined that UFGJ exhibited the highest antioxidant activity. To further investigate the material basis for this elevated antioxidant activity at the metabolite level, we screened the upregulated differential metabolites from the UFGJ vs. FGJ group and the UFGJ vs. GJ group, identifying a total of 323 metabolites after integration (Table S8). Pearson correlation analysis revealed that 289 metabolites displayed significant correlations (P < 0.01) with antioxidant-related indices (TPC, TFC, ABTS^+^, and DPPH), which are hypothesized to be potential key antioxidant metabolites (Table S9 and Fig. 5). These compounds primarily include organic acids and derivatives (e.g., fumaric acid and succinic acid), lipids and lipid-like molecules (e.g., geranyl acetate, 3-hydroxypentanoic acid, and 2-hydroxymyristic acid), organoheterocyclic compounds (e.g., D-glucuronolactone and kojic acid), among others. To further clarify which of these 289 metabolites serve as key antioxidant metabolites and to identify their target mechanisms of action, we performed a network pharmacological analysis to uncover their potential antioxidant mechanisms.

### 3.7 Network pharmacology and molecular docking

#### 3.7.1 Network pharmacology target screening and Construction of a “Flavonoid-Metabolite-Target” network diagram

**Fig. 6:**
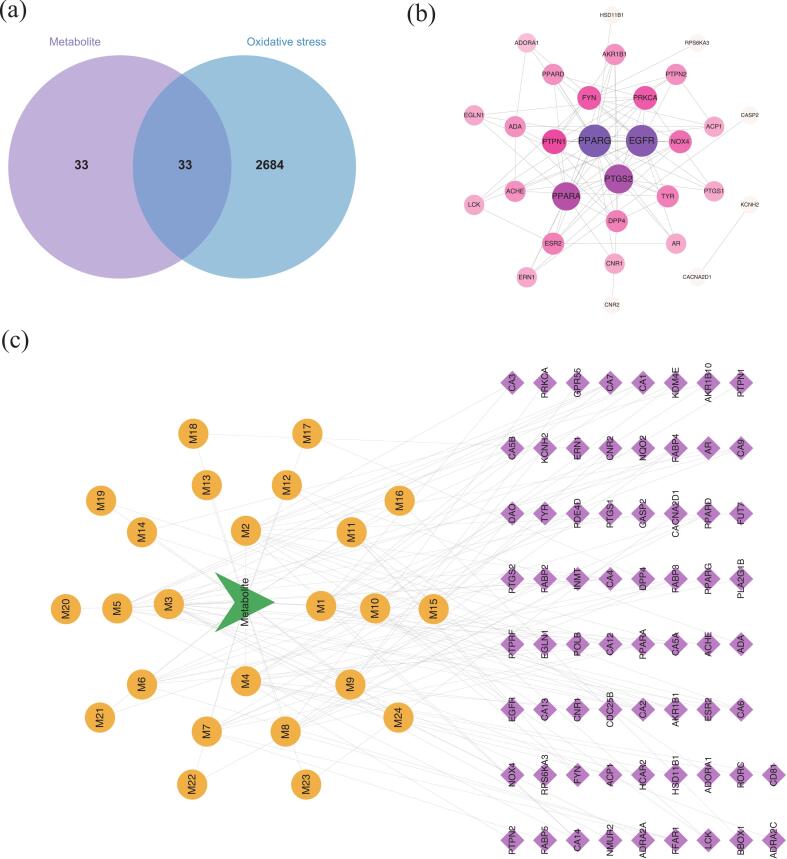
(a) Venn diagram of key antioxidant metabolite targets versus antioxidant-related targets; (b) “protein–protein interactions” network diagram of 66 intersecting antioxidant targets; and (c) “key antioxidant metabolite-specific metabolite-target” network diagram.

To identify key antioxidant metabolites that distinguish between the treatment groups, we utilized the SwissTargetPrediction database. Using a screening criterion of “Possibility > 0.2,” we identified 24 out of 289 potential antioxidant metabolites as significant, yielding a total of 66 potential targets through integration of all metabolite targets. Additionally, we obtained 2,717 antioxidant-related targets from the GeneCards database. By using a Venn diagram to screen the targets of key antioxidant metabolites against antioxidant targets, we identified 33 intersecting targets (Fig. 6a). To explore the relationship between these key antioxidant metabolites and antioxidant targets, we constructed a “key antioxidant metabolite-specific metabolite-target” network diagram (Fig. 6c). The resulting network exhibited 91 nodes (1 key antioxidant metabolite node, 24 specific metabolite nodes, and 66 antioxidant targets) and 133 action associations. Notably, compounds such as 2-hydroxycinnamic acid, gallic acid, myricetin 3-rhamnoside, and ursolic acid displayed multiple connecting lines, suggesting their potential importance in interactions with various targets.

2-Hydroxycinnamic acid, a derivative of hydroxycinnamic acid, exhibits a wide range of biological activities, including antioxidant, anti-inflammatory, bacteriostatic, and antitumor effects [68]. Hydroxycinnamic acid compounds contain a catechol structure, allowing them to react with free radicals to form quinones, thus producing antioxidant effects [65]. Gallic acid is a naturally occurring polyphenolic compound widely present in grapes [21] and tea [33] demonstrating a variety of effects, including antioxidant, anticancer, hypoglycemic, and antimicrobial properties [1]. Furthermore, Wu et al. [86] evaluated the copigmentation effect of gallic acid on red wine color and found that it significantly enhanced both the color quality and stability of red wine. Myricetin 3-rhamnoside, a flavonol found in large quantities in the fruits, barks, and leaves of the Waxaceae family, is known for its hypoglycemic, hypotensive, and anticancer effects [47]. Singh et al. [71] reported that myricetin 3-rhamnoside has an antiproliferative effect on breast cancer cell lines, suggesting it may serve as a drug source to design and synthesize novel and safe treatments for hormone-dependent breast cancer. Ursolic acid, a triterpenoid abundant in apples, grapes, and pears, has demonstrated various beneficial effects in human and animal models, including antioxidant, anti-inflammatory, anti-carcinogenic, and antiviral properties [72].

To screen for core antioxidant targets, we constructed a protein–protein interaction (PPI) network by analyzing the interactions of the 33 intersecting antioxidant targets (Fig. 6b). This network contained 30 nodes and 87 edges, with the top-ranked core genes identified as PPARG, EGFR, PTGS2, and PPARA.

PPARG is a nuclear receptor that binds peroxisome proliferators, such as lipid-lowering drugs and fatty acids, to regulate the peroxisomal oxidation pathway of fatty acids and is involved in biological processes such as adipocyte differentiation and lipid metabolism [55]. Yi et al. [91] found that rutin enhances oxidative stress management by regulating peroxisome proliferator-activated receptor γ (PPARγ). EGFR, a member of the epidermal growth factor receptor family, binds to its ligand AREG at the cell membrane, playing a role in regulating cell survival, proliferation, and motility [82]. Lekmine et al. [44] demonstrated that compounds derived from Hyoscyamus niger express antioxidant effects through EGFR inhibition, predicting their therapeutic potential for targeting ovarian cancer. PTGS2 plays a crucial role in regulating the inflammatory response through prostaglandin production and has been shown to slow the progression of arthritis in mice by downregulating NF-κB, PTGS2, and inhibiting IL-1β, IL-6, IL-23, and IL-17 signaling [26]. PPARA is involved in regulating fatty acid oxidation in various peripheral tissues and plays an important role in physiological processes, including cell differentiation, development, and metabolism [56,97]. Liu et al. [48] found that Pleurotus placentodes significantly decreased the mRNA expression of PTGS2 and increased the mRNA expression of PPARA in liver-injured mice, enhancing antioxidant effects and improving liver function. Through the analysis above, we hypothesize that these target genes play significant roles in the antioxidant effects of key metabolites.

In order to further understand the functional pathways associated with these target genes, further GO enrichment analysis and KEGG pathway enrichment analysis of the target proteins will be required.

#### 3.7.2 GO enrichment analysis and KEGG signaling pathway analysis

**Fig. 7:**
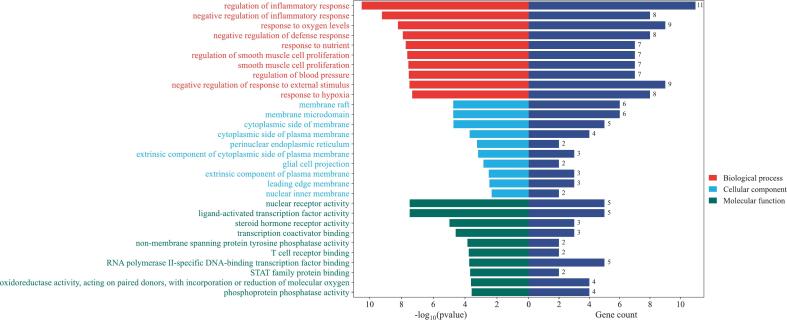
GO enrichment analysis results.

As shown in Table S10, the GO enrichment analysis revealed a total of 2,319 GO entries (Fig. 7). The analysis indicates that biological processes are primarily enriched in the regulation of the inflammatory response, while cellular components are predominantly localized in membrane rafts, and molecular functions are largely centered on nuclear receptor activities.

**Fig. 8:**
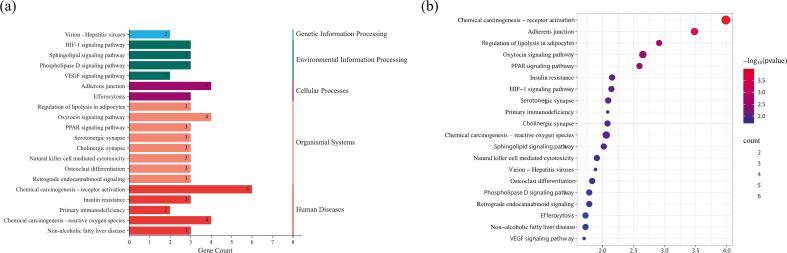
(a) The classification of the top 20 pathways of KEGG pathway enrichment analysis and (b) the dot plot of the top 20 pathways of KEGG pathway enrichment analysis.

The KEGG pathway enrichment analysis identified 33 intersecting targets enriched across 171 pathways (Table S11). As illustrated in Fig. 8, among the 171 pathways, several metabolic pathways—including chemical oncogenic receptor activation, adherens junctions, chemical oncogenic reactive oxygen species, the oxytocin signaling pathway, and the PPAR signaling pathway—were significantly enriched in antioxidant targets.

#### 3.7.3 Molecular docking

To further elucidate the antioxidant mechanisms of key metabolites, including 2-hydroxycinnamic acid, gallic acid, myricetin 3-rhamnoside, and ursolic acid, which exhibited the highest degree values in the protein–protein interaction (PPI) network, these compounds were selected as small molecule ligands along with the targets PPARG, EGFR, PTGS2, and PPARA for docking analysis. A binding energy of ≤ -1.2 kcal/mol indicates spontaneous binding between the ligand and target, while a binding energy of ≤ -5.0 kcal/mol indicates strong binding affinity [94],Zhao et al. [95].

As shown in Fig. S1, the binding energies of all key metabolites docked with the antioxidant core targets ranged from −7.04 to −4.20 kcal/mol, demonstrating that they can bind spontaneously. This suggests that the key metabolites identified during screening possess favorable binding properties with the selected targets, confirming their effectiveness. Among these, Ursolic Acid exhibited the lowest binding energy with EGFR at −7.04 kcal/mol, indicating a strong affinity, while gallic Acid displayed the highest binding energy with PPARG at −4.20 kcal/mol, suggesting a weaker binding affinity. Given that ursolic acid is a pentacyclic triterpene carboxylic acid and gallic Acid contains a single benzene ring, we hypothesize that the ligand’s structural characteristics influence its binding energy. Xiao et al. [87] investigated the binding energies of olfactory receptors with aroma compounds, concluding that the presence of structures such as benzene rings, C=O double bonds, and C=C double bonds can result in π-π and p-π interactions with amino acid residues in benzene rings, thus enhancing binding interactions.

Additionally, a discernible pattern emerges in the binding energies of the key metabolites with the four core targets: the binding energies of the metabolites with EGFR and PPARA are generally lower than those with PPARG and PTGS2, suggesting a higher likelihood of binding to EGFR and PPARA, thereby facilitating antioxidant activity. Further analysis indicates that the binding energies of different metabolites with the same core targets exhibit similar patterns [4], with myricetin 3-rhamnoside and ursolic acid generally demonstrating lower binding energies compared to 2-hydroxycinnamic acid and gallic acid. Consequently, the screened core targets are more likely to bind with myricetin 3-rhamnoside and ursolic acid, enhancing their antioxidant effects. Duan et al. [25] found that various tea seed oil components, including gallic acid, Epigallocatechin, and Puerarin, interact with core targets such as EGFR, AKT, and ESR1, exerting antimicrobial effects by modulating pathways like PI3K-AKT, MAPK, and IL-17. Zhao et al. [93] reported that Dihydromyricetin and myricetin 3-rhamnoside, the primary active compounds in Vine Tea, induce apoptosis in B16F10 cells and exert multi-pathway effects in melanoma prevention and treatment, with TP53, TNF, PPARG, and PTGS2 being main targets of myricetin 3-rhamnoside. Huang et al. [38] utilized network pharmacology to examine the mechanism of action of Ursolic Acid against atherosclerosis, identifying target genes such as STAT3, PPARG, IL-6, and PTGS2 that may be closely associated with its mechanism. The multi-target properties of gallic acid, myricetin 3-rhamnoside, and ursolic acid are evident in both prior research and the present study, demonstrating that a single compound may act on multiple antioxidant targets, which can interact with each other to exert their effects.

However, due to the limitations of network pharmacology and molecular docking, it is necessary to further validate the identified targets and pathways through animal experiments or metagenomics to confirm the efficacy of metabolic molecules in living organisms. Additionally, questions regarding how specific antioxidant metabolites affect target proteins and how multiple targets exchange signals and interact with one another require experimental verification in future studies. Furthermore, sensory attributes constitute an important area of research for fermented juices; therefore, we will also focus on investigating the changes in the characteristic aromas of grape juice fermented with ultrasonic assistance.

## 4 Conclusion

In this study, three treatment groups—UFGJ, FGJ, and GJ—were established to investigate the similarities and differences in the effects of UFGJ and FGJ on the antioxidant activity and metabolites of GJ, utilizing chemical antioxidant assays and a non-targeted metabolomics approach. Network pharmacology and molecular docking analyses were conducted to explore the potential antioxidant mechanisms of key metabolites in UFGJ. Although network pharmacology provides preliminary insights, these findings require further validation. The functional index results indicated that UFGJ significantly enhanced the TPC, TFC, and antioxidant activity of GJ compared to FGJ and GJ (P < 0.001).

Metabolomics analysis revealed highly significant differences in metabolite composition among the three treatment groups (P < 0.001). The primary differential metabolites in both the FGJ vs. GJ and UFGJ vs. GJ groups included organic acids and derivatives, lipids and lipid-like molecules, organoheterocyclic compounds, and organic oxygen compounds. Additionally, several metabolites, such as L-phenylalanine, L-glutamine, betonicine, 2-hydroxycinnamic acid, traumatic acid, and cinnamic acid, were shared as differential metabolites between the two groups, with their content in UFGJ exceeding that in FGJ. This suggests that ultrasound does not destroy or inhibit the metabolic properties of LAB in GJ but rather enhances their metabolic efficiency and increases the content of beneficial metabolites. Notably, metabolites absent in the FGJ group were produced in the UFGJ group, including araloside A, bergenin, and ursolic acid, which may contribute to the higher antioxidant activity of UFGJ compared to FGJ.

In terms of metabolic pathways, all pathways identified in the UFGJ vs. GJ group were also present in the FGJ vs. GJ group, with a greater total metabolite enrichment in the former. Hence, ultrasound-assisted fermentation does not alter the metabolic pathways of LAB in GJ fermentation but does increase metabolite enrichment.

The results from network pharmacology and molecular docking indicated that 24 key antioxidant metabolites in UFGJ, including 2-hydroxycinnamic acid, gallic acid, and myricetin 3-rhamnoside, exert their antioxidant effects primarily by interacting with core target genes such as PPARG, EGFR, PTGS2, and PPARA. The antioxidant effects of these target genes are mainly executed by regulating signaling pathways, including chemical carcinogenesis—receptor activation, adherens junctions, and PPAR signaling pathways. Moreover, key antioxidant metabolites exhibited favorable binding properties to the selected targets, with ursolic acid demonstrating the most stable binding to EGFR. Future studies should construct models using cell lines, Hidradenitis elegans nematodes, or zebrafish, and integrate transcriptomic and genomic technologies to analyze the impact of key antioxidant metabolites on the expression of related antioxidant enzymes and signaling factors, thus validating their mechanisms of action in UFGJ.

## CRediT authorship contribution statement

**Liyue Fei:** Writing – review & editing, Writing – original draft, Investigation, Formal analysis. **Dongsheng Zhang:** Validation, Supervision, Project administration, Investigation. **Yu Li:** Methodology, Conceptualization. **Johane Johari Mkunga:** Methodology, Conceptualization. **Zinan Zhang:** Validation, Investigation, Data curation. **Chenglong He:** Methodology, Conceptualization. **Chunhui Shan:** Supervision, Software, Data curation. **Muhammad Iqbal Choudhary:** Writing – review & editing. **Xinquan Yang:** Project administration. **Wenchao Cai:** Writing – review & editing, Supervision, Project administration, Funding acquisition.

## Declaration of competing interest

The authors declare that they have no known competing financial interests or personal relationships that could have appeared to influence the work reported in this paper.

## References

[1] Baite T.N., Mandal B., Purkait M.K. (2021). Ultrasound assisted extraction of gallic acid from Ficus auriculata leaves using green solvent. Food Bioprod. Process..

[2] Bhargava N., Mor R.S., Kumar K., Sharanagat V.S. (2021). Advances in application of ultrasound in food processing: a review. Ultrason. Sonochem..

[3] Blaszak M., Lachowicz-Wisniewska S., Kapusta I., Szewczuk M., Ochmian I. (2025). Enhanced Extraction of Polyphenols, Physicochemical Properties, and Microbial Control in Vitis vinifera L. juice using Ultrasound-Assisted Maceration. Molecules.

[4] Cai W., Chen P., Zhang D., Mkunga J.J., Fei L., He C., Ma C. (2025). Enhancing jujube juice quality through Lactiplantibacillus plantarum fermentation: a metabolomics approach. Food Chem..

[5] Cai W., Tang F., Wang Y., Zhang Z., Xue Y., Zhao X., Shan C. (2021). Bacterial diversity and flavor profile of Zha-Chili, a traditional fermented food in China. Food Res. Int..

[6] Cai W., Wang Y., Hou Q., Zhang Z., Tang F., Shan C., Guo Z. (2021). PacBio sequencing combined with metagenomic shotgun sequencing provides insight into the microbial diversity of zha-chili. Food Biosci..

[7] Cai W., Wang Y., Hou Q., Zhang Z., Tang F., Shan C., Guo Z. (2021). Rice varieties affect bacterial diversity, flavor, and metabolites of zha-chili. Food Res. Int..

[8] Cai W., Wang Y., Liu Z., Liu J., Zhong, J.a., Hou, Q., & Guo, Z. (2022). Depth-depended quality comparison of light-flavor fermented grains from two fermentation rounds. Food Research International (ottawa, Ont.).

[9] Cai W.C., Fei L.Y., Zhang D.S., Mao Y.W., Lu Z.Y., Li Y., Zheng Q.Q. (2025). Impact of Lactobacillus plantarum fermentation on jujube juice: Insights from intelligent sensory evaluation, tolerance index, and acidogenic potential. Lwt-Food Science and Technology.

[10] Cai W.C., Fei L.Y., Zhang D.S., Ni H., Peng B., Zhao X.X., Shan C.H. (2024). Impact of ultra-high-pressure treatment on microbial community composition and flavor quality of jujube juice: Insights from high-throughput sequencing technology, intelligent bionic sensory system, and metabolomics approach. Food Res. Int..

[11] Cai W.C., Tang F.X., Guo Z., Guo X., Zhang Q., Zhao X.X., Shan C.H. (2020). Effects of pretreatment methods and leaching methods on jujube wine quality detected by electronic senses and HS-SPME–GC–MS. Food Chem..

[12] Cai W.C., Tang F.X., Shan C.H., Hou Q.C., Zhang Z.D., Dong Y., Guo Z. (2020). Pretreatment methods affecting the color, flavor, bioactive compounds, and antioxidant activity of jujube wine. Food Sci. Nutr..

[13] Cai W.C., Tang F.X., Shan C.H., Yang L.P., Zhan Q., Zhao X.X., Ning M. (2018). Changes in volatile compounds of fermented mixed drink using commercial yeast strain. Przem Chem.

[14] Cai W.C., Tang F.X., Zhao X.X., Guo Z., Zhang Z.D., Dong Y., Shan C.H. (2019). Different lactic acid bacteria strains affecting the flavor profile of fermented jujube juice. J. Food Process. Preserv..

[15] Cai W.C., Wang Y.R., Ni H., Liu Z.J., Liu J.M., Zhong J.A., Guo Z. (2021). Diversity of microbiota, microbial functions, and flavor in different types of low-temperature Daqu. Food Res. Int..

[16] Chen B., Lu N., Lee K., Ye L., Hasegawa C., Maeda K. (2022). Application of mevalonolactone prevents deterioration of epidermal barrier function by accelerating the lamellar granule lipid transport system. Skin Res. Technol..

[17] Chen J.L., Wang Q.Q., Wu Y.T., Wu Y., Sun Y., Ding Y.F., Tao Y. (2023). Ultrasound-assisted fermentation of ginkgo kernel juice by Lactiplantibacillus plantarum: Microbial response and juice composition development. Ultrason. Sonochem..

[18] Chen M., Zhang S.N., Huang X.Q., Zhang D.D., Zhu D., Ouyang C.H., Li Y.K. (2025). The protective effects and mechanism of myricetin in liver diseases (Review). Mol. Med. Rep..

[19] Chen Y., Jiang J., Li Y., Xie Y., Cui M., Hu Y., Cheng W. (2024). Enhancing physicochemical properties, organic acids, antioxidant capacity, amino acids and volatile compounds for ‘Summer Black’grape juice by lactic acid bacteria fermentation. LWT.

[20] Chen Y.W., Jiang J.Q., Li Y.K., Xie Y., Cui M., Hu Y., Gao F.F. (2024). Enhancing physicochemical properties, organic acids, antioxidant capacity, amino acids and volatile compounds for 'Summer Black' grape juice by lactic acid bacteria fermentation. Lwt-Food Science and Technology.

[21] Chikere C.O., Hobben E., Faisal N.H., Kong-Thoo-Lin P., Fernandez C. (2021). Electroanalytical determination of gallic acid in red and white wine samples using cobalt oxide nanoparticles-modified carbon-paste electrodes. Microchem. J..

[22] Chung D., Kim S.Y., Ahn J.-H. (2017). Production of three phenylethanoids, tyrosol, hydroxytyrosol, and salidroside, using plant genes expressing in Escherichia coli. Sci. Rep..

[23] Dai Z., Deng K.L., Wang X.M., Yang D.X., Tang C.L., Zhou Y.P. (2024). Bidirectional effects of the tryptophan metabolite indole-3-acetaldehyde on colorectal cancer. *World Journal of*. Gastrointest. Oncol..

[24] Dong Y.J., Wu Y.X., Zhang Z.X., Wang S.C., Cheng J., Gao Y.L., Wang Y.X. (2023). Transcriptomic analysis reveals GA3 is involved in regulating flavonoid metabolism in grape development for facility cultivation. Mol. Genet. Genomics.

[25] Duan Y., Zhao L.J., Zhou Y.H., Zhou Q.Z., Fang A.Q., Huang Y.T., Li J. (2023). UPLC-Q-TOF-MS, network analysis, and molecular docking to investigate the effect and active ingredients of tea-seed oil against bacterial pathogens. Front. Pharmacol..

[26] El-Tanbouly G.S., Abdelrahman R.S. (2022). Novel anti-arthritic mechanisms of trans-cinnamaldehyde against complete Freund’s adjuvant-induced arthritis in mice: involvement of NF-кB/TNF-α and IL-6/IL-23/IL-17 pathways in the immuno-inflammatory responses. Inflammopharmacology.

[27] Eniafe J., Jiang S. (2021). The functional roles of TCA cycle metabolites in cancer. Oncogene.

[28] Fang Z.Z., Lin-Wang K., Lin Y.J., Espley R.V. (2025). Metabolomic and transcriptomic analyses provide insights into temperature and light regulated anthocyanin accumulation in flesh of 'Furongli' plum (Prunus salicina Lindl.). Postharvest Biol. Technol..

[29] Fei L., Zhang D., Mao Y., Mkunga J.J., Chen P., He C., Cai W. (2025). Metabolomics combined with network pharmacology reveals the regional and variety heterogeneity of grape metabolites and their potential antioxidant mechanisms. Food Res. Int..

[30] Fitzpatrick P.F. (2023). The aromatic amino acid hydroxylases: Structures, catalysis, and regulation of phenylalanine hydroxylase, tyrosine hydroxylase, and tryptophan hydroxylase. Arch. Biochem. Biophys..

[31] Gao Z.X., Zhang Z.S., Qin J., Zhang M.Z., Cao J.L., Li Y.Y., Xie S.Q. (2023). Aucubin enhances the antitumor activity of cisplatin through the inhibition of PD-L1 expression in hepatocellular carcinoma. Phytomedicine.

[32] Gouot J.C., Smith J.P., Holzapfel B.P., Walker A.R., Barril C. (2019). Grape berry flavonoids: a review of their biochemical responses to high and extreme high temperatures. J. Exp. Bot..

[33] Gutierrez A.S., Guo J.Y., Feng J.N., Tan L.B., Kong L.Y. (2020). Inhibition of starch digestion by gallic acid and alkyl gallates. Food Hydrocoll..

[34] Hashemi S.M.B., Jafarpour D., Soto E.R., Barba F.J. (2023). Ultrasound-Assisted Lactic Acid Fermentation of Bakraei (Citrus reticulata cv. Bakraei) Juice: Physicochemical and Bioactive Properties. Fermentation-Basel.

[35] He C.L., Mkunga J.J., Zhang D.S., Mao Y.W., Fei L.Y., Chen P.P., Cai W.C. (2025). Integrative analysis of the fermentation mechanisms, antioxidant properties, and potential health benefits of mulberry wine using multiple bioinformatics approaches. Food Biosci..

[36] He C.L., Peng B., Zhang D.S., Fei L.Y., Mao Y.W., Lu Z.Y., Cai W.C. (2025). Comparative analysis of strong-flavor Baijiu and brandy: volatile and nonvolatile metabolic compounds identified using non-targeted metabolomics. Eur. Food Res. Technol..

[37] He C.L., Zhang D.S., Mao Y.W., Mkunga J.J., Fei L.Y., Chen P.P., Cai W.C. (2025). Integrative analysis of metabolite changes and potential health effects in pomegranate juice fermentation. Food Biosci..

[38] Huang N., Xing Q.C., Li W.C., Yan Q.Z., Liu R.Z., Liu X., Liu Z. (2023). Explore the mechanism of ursolic acid acting on atherosclerosis through network pharmacological and bioinformatics methods. Medicine.

[39] Jadhav U., Mundhe S., Kumar Y., Jogaiah S., Upadhyay A., Gupta V.S., Kadoo N.Y. (2021). Gibberellic Acid Induces Unique Molecular responses in 'Thompson Seedless' grapes as Revealed by Non-targeted Metabolomics. J. Plant Growth Regul..

[40] Kartini K., Irawan M.A., Setiawan F., Jayani N.I.E. (2023). Characteristics, Isolation Methods, and Biological Properties of Aucubin. Molecules.

[41] Kaynarca G.B., Kamer D.D.A., Yucel E., Yilmaz O.S., Henden Y., Kaymaz E., Gumus T. (2024). The potential of pectin-based films enriched with bioactive components for strawberry preservation: a sustainable and innovative coating. Sci. Hortic..

[42] Kuerban D., Lu J., Huangfu Z.K., Wang L., Qin Y.A., Zhang M.W. (2023). Optimization of Fermentation Conditions and Metabolite Profiling of Grape juice Fermented with Lactic Acid Bacteria for improved Flavor and Bioactivity. Foods.

[43] Lazzara F., Conti F., Giuffrida E., Eandi C.M., Drago F., Platania C.B.M., Bucolo C. (2024). Integrating network pharmacology: the next-generation approach in ocular drug discovery. Curr. Opin. Pharmacol..

[44] Lekmine S., Benslama O., Kadi K., Martin-Garcia A.I., Ola M.S., Yilmaz M.A., Ali A. (2024). Therapeutic potential of *Hyoscyamus* niger-derived compounds: Targeting ovarian cancer through antioxidant activity and EGFR tyrosine kinase inhibition. *Journal of King Saud University*. Science.

[45] Liang S.H., Liu Y.M., Meng Y.N., Sun C. (2021). Two-stage Enzymatic Hydrolysis of soybean Concentrated Phospholipid to Prepare Glycerylphosphorylcholine: Optimized by Response Surface Methodology. J. Oleo Sci..

[46] Liao L., Tang Y., Li B., Tang J., Xu H., Zhao K., Zhang X.C. (2023). Stachydrine, a potential drug for the treatment of cardiovascular system and central nervous system diseases. Biomed. Pharmacother..

[47] Liu K.F., Zhang Y.B., Zhang W., Liu L.Y., Yu Z. (2023). A Study on the Interactions of Proteinase K with Myricetin and Myricitrin by Multi-Spectroscopy and Molecular Modeling. Int. J. Mol. Sci..

[48] Liu X.-Q., Shao W.-J., Liu X.-P., Zhang Y., Liu H., Wang J.-M., Ma C. (2024). Protective effects of *Pleurotus placentodes* against liver injury in mice via the PTGS2, NR3C1 and PPARA signaling pathways. J. Funct. Foods.

[49] Liu Y., Zhu J., Zhu C. (2024). Effect of ultrasonic pretreatment on fermentation performance and quality of fermented hawthorn pulp by lactic acid bacteria. Food Chem..

[50] Liu Y., Zhu J.X., Zhu C.H. (2024). Effect of ultrasonic pretreatment on fermentation performance and quality of fermented hawthorn pulp by lactic acid bacteria. Food Chem..

[51] Liu Y.Y., Tan Y.Z., Cao G.J., Shi L., Song Y.J., Shan W.J., Yi W. (2023). Bergenin alleviates myocardial ischemia-reperfusion injury via SIRT1 signaling. Biomed. Pharmacother..

[52] Liu Z., Huang X.Z., Liu Q.Z., Yang J.H., Li J.H., Xiao M.Y., Xie M.Y. (2025). Lactic acid bacteria fermentation improves sensory properties, bioactivity, and metabolic profiles of carrot pulp. Food Biosci..

[53] Luo Y., Tang R.L., Qiu H., Song A.X. (2024). Widely targeted metabolomics-based analysis of the impact of L. plantarum and L. paracasei fermentation on rosa roxburghii Tratt juice. Int. J. Food Microbiol..

[54] Ma J.H., Wang J., Wang J.Q., Zhou J., Jiang C.H., Chen W.H., Chen M.W. (2024). Araloside a alleviates sepsis-induced acute lung injury via PHD2/HIF-1α α in macrophages. Phytomedicine.

[55] Miao Y., Wu X., Xue X., Ma X., Yang L., Zeng X., Wei Z. (2023). Morin, the PPARγ agonist, inhibits Th17 differentiation by limiting fatty acid synthesis in collagen-induced arthritis. Cell Biol. Toxicol..

[56] Montaigne D., Butruille L., Staels B. (2021). PPAR control of metabolism and cardiovascular functions. Nat. Rev. Cardiol..

[57] Moutiez M., Belin P., Gondry M. (2017). Aminoacyl-tRNA-utilizing Enzymes in Natural Product Biosynthesis. Chem. Rev..

[58] Mukhopadhyay A., Borkakoti N., Pravda L., Tyzack J.D., Thornton J.M., Velankar S. (2019). Finding enzyme cofactors in Protein Data Bank. Bioinformatics.

[59] Narukawa M., Kamiyoshihara A., Izu H., Fujii T., Matsubara K., Misaka T. (2020). Efficacy of Long-Term Feeding of α-Glycerophosphocholine for Aging-Related Phenomena in Old mice. Gerontology.

[60] Nery-Flores S.D., Castro-Lopez C.M., Martinez-Hernandez L., Garcia-Chavez C.V., Palomo-Ligas L., Ascacio-Valdes J.A., Rodriguez-Herrera R. (2024). Grape Pomace Polyphenols Reduce Acute Inflammatory Response Induced by Carrageenan in a Murine Model. Chem. Biodivers..

[61] Pawar S.V., Rathod V.K. (2020). Role of ultrasound in assisted fermentation technologies for process enhancements. Prep. Biochem. Biotechnol..

[62] Peng B., Dai Q.H., Liu X.D., Jiang S.Y. (2024). Fraxin alleviates oral lichen planus by suppressing OCT3-mediated activation of FGF2/NF-κB pathway. Naunyn-Schmiedebergs Archives of Pharmacology.

[63] Ranjbaran E., Gholami M., Jensen M. (2021). Changes in phenolic compounds, enzymatic and non-enzymatic antioxidant properties in “Thompson Seedless” grape after UV-C irradiation. J. Food Process. Preserv..

[64] Rosa L.S., Santos M.L., Abreu J.P., Rocha R.S., Esmerino E.A., Freitas M.Q., Teodoro A.J. (2023). Probiotic fermented whey-milk beverages: effect of different probiotic strains on the physicochemical characteristics, biological activity, and bioactive peptides. Food Res. Int..

[65] Ruwizhi N., Aderibigbe B.A. (2020). Cinnamic Acid Derivatives and their Biological Efficacy. Int. J. Mol. Sci..

[66] Saidi L., Duanis-Assaf D., Galsarker O., Maurer D., Alkan N., Poverenov E. (2021). Elicitation of fruit defense response by active edible coatings embedded with phenylalanine to improve quality and storability of avocado fruit. Postharvest Biol. Technol..

[67] Schenck C.A., Maeda H.A. (2018). Tyrosine biosynthesis, metabolism, and catabolism in plants. Phytochemistry.

[68] Shang Y.J., Liu B.Y., Zhao M.M. (2015). Details of the Antioxidant Mechanism of Hydroxycinnamic Acids. Czech J. Food Sci..

[69] Sharma P., Kashyap P., Dhakane A. (2023). Exploring the antioxidant potential of fermented turmeric pulp: effect of extraction methods and microencapsulation. Prep. Biochem. Biotechnol..

[70] Sheng J., Shan C.H., Liu Y.Y., Zhang P.L., Li J.J., Cai W.C., Tang F.X. (2022). Comparative evaluation of the quality of red globe grape juice fermented by Lactobacillus acidophilus and Lactobacillus plantarum. Int. J. Food Sci. Technol..

[71] Singh S., Maurya A.K., Meena A., Mishra N., Luqman S. (2023). Myricetin 3-rhamnoside retards the proliferation of hormone-independent breast cancer cells by targeting hyaluronidase. Curr. Sci..

[72] Son J., Lee S.Y. (2020). Therapeutic potential of Ursonic Acid: Comparison with Ursolic Acid. Biomolecules.

[73] Song X.M.T., Tan L., Wang M., Ren C.X., Guo C.J., Yang B., Pei J. (2021). Myricetin: a review of the most recent research. Biomed. Pharmacother..

[74] Sun L., Liu L.P., Wang Y.Z., Yang L., Zhang C.S., Yue M.X., Wang L. (2022). Effect of ultrasonication on the metabolome and transcriptome profile changes in the fermentation of Ganoderma lucidum. Microbiol. Res..

[75] Sun Y.Q., Liang M.M., Xing Y.M., Duan Y.F., Zhang S.X., Deng B.G., Zhou B.E. (2023). Cyasterone has a protective effect on steroid-induced Osteonecrosis of the femoral head. PLoS One.

[76] Takahashi H., Nishitani K., Kawarasaki S., Martin-Morales A., Nagai H., Kuwata H., Goto T. (2024). Metabolome analysis reveals that cyclic adenosine diphosphate ribose contributes to the regulation of differentiation in mice adipocyte. FASEB J..

[77] Takénaka A., Moras D. (2020). Correlation between equi-partition of aminoacyl-tRNA synthetases and amino-acid biosynthesis pathways. Nucleic Acids Res..

[78] Tang Z.Z., Zhao Z.Q., Chen S.Y., Lin W.J., Wang Q., Shen N.Y., Yuan M. (2023). Dragon fruit-kiwi fermented beverage: *In vitro* digestion, untargeted metabolome analysis and anti-aging activity in *Caenorhabditis elegans*. Front. Nutr..

[79] Tian Y.Q., Ou Z.Y., Li F.M., Fan W.G., Ren H.W., Yang W.X., Zhang X.L. (2023). Synergistic protection by Araloside a with L-ascorbic acid on oxidative stress via Nrf2/CAT activation in HEK293 cells. J. Funct. Foods.

[80] Wang H.M., Tao Y., Li Y.T., Wu S.S., Li D.D., Liu X.W., Show P.L. (2021). Application of ultrasonication at different microbial growth stages during apple juice fermentation by Lactobacillus plantarum: Investigation on the metabolic response. Ultrason. Sonochem..

[81] Wang H.N., Mei L., Deng Y., Liu Y.H., Wei X.Q., Liu M., Li M. (2019). Lactobacillus brevis DM9218 ameliorates fructose-induced hyperuricemia through inosine degradation and manipulation of intestinal dysbiosis. Nutrition.

[82] Wang J., Cui W., Dou X., Yin B., Niu Y., Niu L., Yan G. (2024). Euonymus alatus delays progression of diabetic kidney disease in mice by regulating EGFR tyrosine kinase inhibitor resistance signaling pathway. *Nan fang yi ke da xue xue bao =*. Journal of Southern Medical University.

[83] Wang M.M., Maeda H.A. (2018). Aromatic amino acid aminotransferases in plants. Phytochem. Rev..

[84] Wang W.T., Mao J.Y., Chen J.Y., Huang W.Y., Duan T.T., Zhang Y., Wu X.M. (2025). Metabolic differences and potential action mechanisms of core antioxidant substances of Gastrodia elata in Guizhou Province, China by untargeted metabolomics and network pharmacology. Food Biosci..

[85] Wu B.M., Liu J.C., Yang W.B., Zhang Q., Yang Z.Y., Liu H., Jiao Z.G. (2021). Nutritional and flavor properties of grape juice as affected by fermentation with lactic acid bacteria. Int. J. Food Prop..

[86] Wu L.L., Zhang Y., Prejanò M., Marino T., Russo N., Tao Y.S., Li Y.K. (2024). Gallic acid improves color quality and stability of red wine via physico-chemical interaction and chemical transformation as revealed by thermodynamics, real wine dynamics and benchmark quantum mechanical calculations. Food Res. Int..

[87] Xiao Z.B., Shen T.Y., Ke Q.F., Shen X.J., Yang E.Q., Sun Z.C., Zhu J.C. (2024). Identification of characteristic aroma compounds of Longjing tea and their molecular mechanisms of interaction with olfactory receptors using molecular docking. Eur. Food Res. Technol..

[88] Yang L., Zheng Y., Miao Y.M., Yan W.X., Geng Y.Z., Dai Y., Wei Z.F. (2022). Bergenin, a PPARγ agonist, inhibits Th17 differentiation and subsequent neutrophilic asthma by preventing GLS1-dependent glutaminolysis. Acta Pharmacol. Sin..

[89] Yao H.Y., Zhao J.Q., Song X.Y. (2022). Protective effects of fraxin on cerebral ischemia-reperfusion injury by mediating neuroinflammation and oxidative stress through PPAR-?/NF-?B pathway. Brain Res. Bull..

[90] Yao L.L., Liu W., Bashir M., Nisar M.F., Wan C.P. (2022). Eriocitrin: a review of pharmacological effects. Biomed. Pharmacother..

[91] Yi T., Zhang W., Hua Y., Xin X., Wu Z., Li Y., Xu L. (2024). Rutin alleviates lupus nephritis by inhibiting T cell oxidative stress through PPARγ. Chem. Biol. Interact..

[92] Zhao, L.,Maimaitiyiming, R.,Hong, J.Y.,Wang, L.,Mu, Y.,Liu, B.Z., & Aihaiti, A. (2024a). Optimization of tomato (Solanum lycopersicum L.) juice fermentation process and analysis of its metabolites during fermentation (vol 11, 1344117, 2024). *Frontiers in Nutrition, 11*. https://doi.org/10.3389/fnut.2024.1423909. 10.3389/fnut.2024.1344117 PMC10868405 38362104

[93] Zhao N.X., Kong H.M., Liu H.S., Shi Q., Qi X.Y., Chen Q.P. (2022). A network pharmacology approach to evaluate the synergistic effect of dihydromyricetin and myricitrin in vine tea on the proliferation of B16F10 cells. Front. Nutr..

[94] Zhao Q., Li Z., Zhang K., Deng X., Wang G., Ye Z., Ye X. (2025). Revealing the off-flavors in hydro-distilled essential oils of sweet orange (Citrus sinensis) by flavoromics strategy and computational simulation. Food Chem..

[95] Zhao X., Wang Z., Tang F., Cai W., Peng B., Shan C. (2024). Exploring jujube wine flavor and fermentation mechanisms by HS-SPME-GC–MS and UHPLC-MS metabolomics. Food Chem.: X.

[96] Zhao Y., He W., Zhan P., Geng J., Wang P., Tian H. (2024). A comprehensive analysis of aroma quality and perception mechanism in ginger-infused stewed beef using instrumental analysis, sensory evaluation and molecular docking. Food Chem..

[97] Zhou B., Zhao G., Li H., Zhao Q., Liu D. (2023). FNDC5/PPARa Pathway Alleviates THP-1-derived Macrophage Pyroptosis and its Mechanism. Altern. Ther. Health Med..

