# Supplementary figures and images for: Metabolomic insights into ultrasound-assisted fermentation of grape juice
Source: Ultrason Sonochem. 2025 Aug 30;121:107537. doi: 10.1016/j.ultsonch.2025.107537 (PMC12423418; doi:10.1016/j.ultsonch.2025.107537)

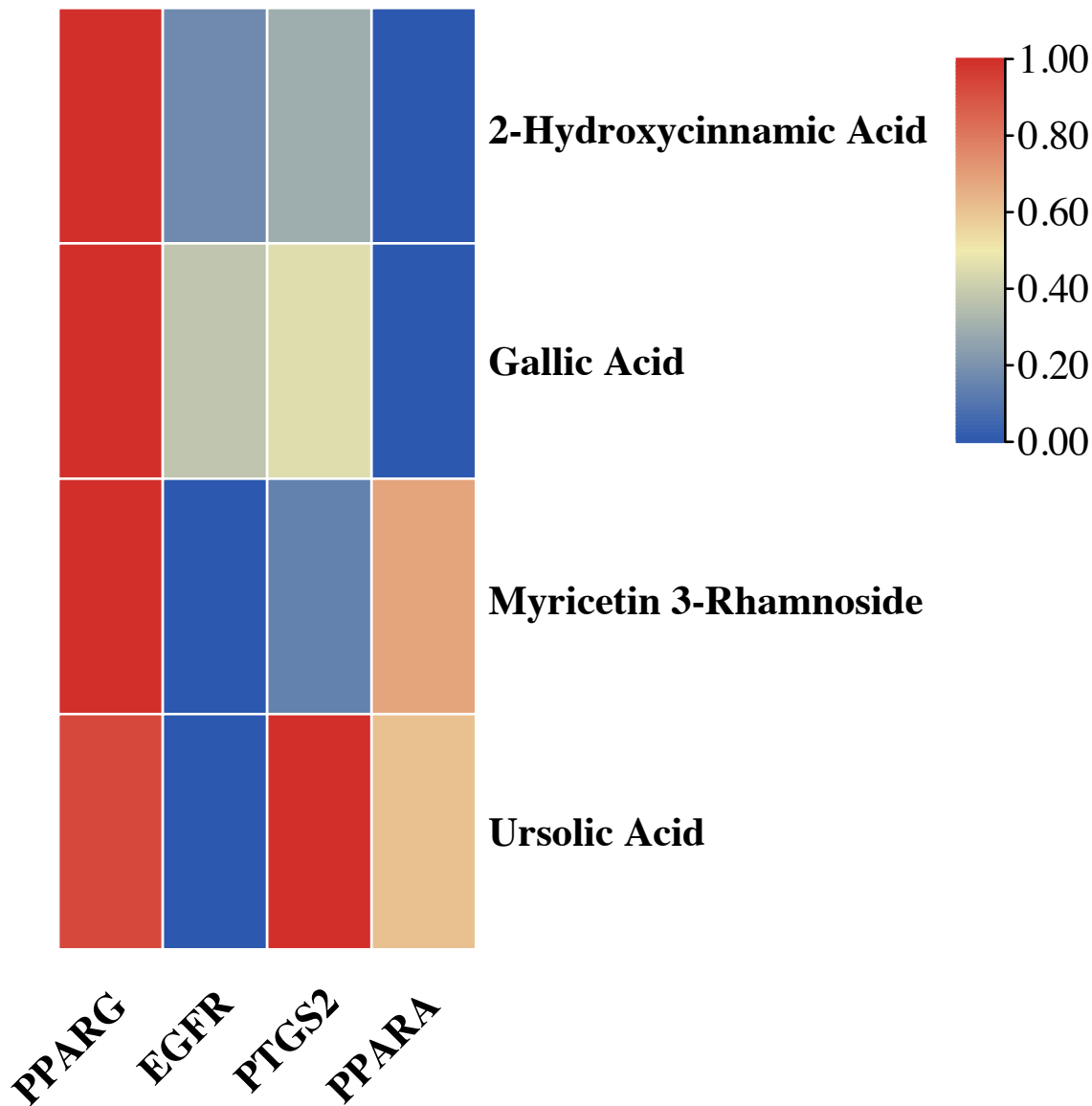

Supplement: Supplementary Data 1 [file mmc1.pdf]
